# Hepatocellular Carcinoma, Alpha Fetoprotein, and Liver Allocation for Transplantation: Past, Present and Future

**DOI:** 10.3390/curroncol29100593

**Published:** 2022-10-08

**Authors:** Brianna Ruch, Josiah Wagler, Kayla Kumm, Chi Zhang, Nitin N. Katariya, Mauricio Garcia-Saenz-de-Sicilia, Emmanouil Giorgakis, Amit K. Mathur

**Affiliations:** 1 Department of Surgery, Division of Transplant Surgery, Mayo Clinic, Phoenix, AZ 85054, USA; 2 Department of Surgery, Mayo Clinic Arizona, Phoenix, AZ 85054, USA; 3 Robert D. and Patricia E. Kern Center for the Science of Health Care Delivery, Mayo Clinic, Rochester, MN 55905, USA; 4 Department of Internal Medicine, Division of Gastroenterology and Hepatology, University of Arkansas for Medical Sciences, Little Rock, AR 72205, USA; 5 Department of Surgery, Division of Solid Organ Transplant, University of Arkansas for Medical Sciences Medical Center, Little Rock, AR 72205, USA

**Keywords:** hepatocellular carcinoma, alpha fetoprotein, liver transplantation, liver allocation

## Abstract

Hepatocellular carcinoma (HCC) is one of the leading indications for liver transplantation and has been the treatment of choice due to the oncologic benefit for patients with advanced chronic liver disease (AdvCLD) and small tumors for the last 25 years. For HCC patients undergoing liver transplantation, alpha fetoprotein (AFP) has increasingly been applied as an independent predictor for overall survival, disease free recurrence, and waitlist drop out. In addition to static AFP, newer studies evaluating the AFP dynamic response to downstaging therapy show enhanced prognostication compared to static AFP alone. While AFP has been utilized to select HCC patients for transplant, despite years of allocation policy changes, the US allocation system continues to take a uniform approach to HCC patients, without discriminating between those with favorable or unfavorable tumor biology. We aim to review the history of liver allocation for HCC in the US, the utility of AFP in liver transplantation, the implications of weaving AFP as a biomarker into policy. Based on this review, we encourage the US transplant community to revisit its HCC organ allocation model, to incorporate more precise oncologic principles for patient selection, and to adopt AFP dynamics to better stratify waitlist dropout risk.

## 1. Background

Hepatocellular carcinoma (HCC) comprises 80% of primary liver cancers [1,2]. It has an average five-year disease specific survival of only 21.5% [3] and is the leading cause of death in patients with compensated cirrhosis [4]. It is currently the third most common indication for liver transplantation within the United States [5].

HCC management is often complicated by concomitant liver disease and cirrhosis, making surgical resection for locoregional control implausible for many patients [2]. In 1996, Mazzaferro et al. found liver transplantation provided an oncologic benefit for patients with small HCCs, and established the Milan Criteria, which precipitated the mainstream adoption of liver transplant as a viable HCC treatment option for a subset of patients [6].

Despite the adoption of the Milan Criteria in 1997, the United States transplant allocation model for HCC has required numerous revisions to address difficulties in appropriate listing. These revisions aimed to equably list HCC patients alongside cirrhotic patients, while minimizing waitlist drop off and post-transplant recurrence. The initial allocation guidelines and subsequent revisions were imaging-based. Over time, imaging prediction models were found to be subject to inaccuracies when determining the extent of tumor burden and predicting tumor aggressiveness [7].

**Figure 1:**
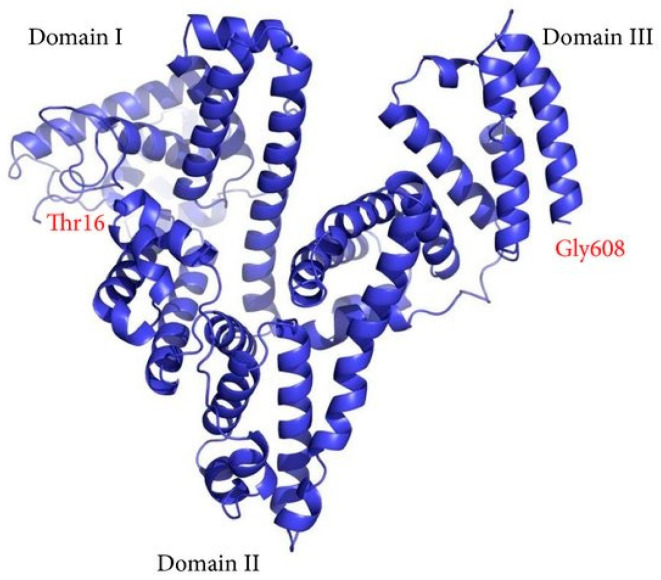
AFP structure model. Copyright^© 2022^ Mingyue Zhu et al. is licensed under CC BY 3.0 [11].

Alpha fetoprotein (AFP) is a major mammalian embryo-specific and tumor-associated glycoprotein made of 591 amino acids and a carbohydrate moiety [8,9,10]. It has a V-shaped structure comprised of three major domains, with the active binding sites in domain I and III, Figure 1 [11]. It is encoded by the AFP gene on chromosome 4q25 and largely produced by the embryonic yolk sac and liver during fetal development [9]. Small amounts of AFP may also be present in adults under normal conditions [12]. Elevated levels have been used as a screening tool in congenital abnormalities, chromosomal disease, and malignancies, including HCC [9]. AFP has increasingly been found to serve as a predictor of HCC-related liver transplant patient survival, HCC tumor recurrence, and waitlist drop out [13,14,15]. Although the role of AFP in liver transplantation has been reviewed [4,16], there is not a centralized discussion of how AFP has been incorporated into allocation, or the new potential roles of AFP dynamics and waitlist stratification. The aim of this review was to provide an evolutionary perspective of liver transplantation and allocation for HCC, focusing on the increasingly central role of AFP as an HCC biomarker, and future directions.

## 2. History of Liver Allocation Policy for Transplantation for HCC within the United States 

Initial transplantation results for HCC were lamentable, with a patient survival of only 20–40 percent at five years [6,17]. The Milan criteria were established utilizing imaging criteria (largest tumor < 5 cm, or no more than 3 tumor nodules, each <3 cm and no obvious vessel or nodal involvement) [6]. The initial Mazzafero study is widely acknowledged as the first demonstration of a survival benefit for transplanting patients with small HCCs. The oncologic benefit identified in this study was clear–liver transplantation provided 75% overall and 83% recurrence-free survival at 4 years [6]. This opened the avenue for HCC to become a viable indication for liver transplantation. There were rapidly calls for the creation and inclusion of HCC criteria in the United States liver allocation policy.

In 1997, the liver allocation system in the United States was based on the assignment of the Child-Turcotte-Pugh (CTP) score and patients assigned to one of four categories when wait-listed: Status 1, Status 2A, Status 2B and Status 3. Unlike patients with decompensated cirrhosis who were wait-listed, HCC patients often retained hepatic synthetic function and would not mount a CPT score that was competitive for transplantation. As a means to prevent patient dropout from disease advancement past Milan Criteria, all listed HCC patients were given Status 2B if their disease fell within Milan Criteria [17]. 

In 2002, liver allocation models shifted. The Model for End-Stage Liver Disease (MELD) replaced CTP as the scoring system for liver allocation, following a Malinchoc et al. study showing better prediction of short-term survival [18,19]. This model retained the principle of transplanting the sickest patients first but utilized objective measures of liver dysfunction that were less vulnerable to gaming. While MELD-based allocation brought greater objectivity in establishing priority on the liver transplant waiting list, gaps remained for those whose survival was not accurately predicted by the biochemical values of MELD components (INR, bilirubin, and creatinine). For patients with HCC, many of whom were listed for transplant with normal or near-normal laboratory MELD scores, MELD-based allocation was insufficient in addressing their waitlist mortality risk. To compensate, a MELD exception point scheme was developed to base liver allocation for HCC patients based on stage. This scheme was a MELD point ladder—if patients stayed within Milan Criteria, additional exception points were awarded every 3 months to address the potential increase in mortality HCC patients faced while accruing time on the waitlist. This allocation model subsequently raised concerns regarding the potential over-prioritization of HCC patients on the waitlist, with 86% of HCC stage 1 and 91% HCC stage 2 being transplanted in the first 3 months following listing [17]. In response to these concerns, policy changes were progressively made over time, with lower and lower priority given to MELD exceptions for HCC, in an attempt to create a more equitable balance for waitlisted patients [17,20]. Importantly, allocation policy did not account at that time for effects of liver-directed locoregional therapy such as embolization, chemoembolization, ablation, or other modalities. 

In 2005, Merion et al. reported a survival benefit in patients receiving transplant when MELD > 15 [21]. To direct organs to patients who would sustain the greatest benefit and minimize geographical discrepancies, the US liver distribution system enacted a Regional “Share 15” system. This policy promoted regional sharing of organs for waitlist patients with MELDs > 15 prior to being offered to local patients with MELD < 15 [22]. The MELD ladder system remained in place for MELD exception patients waitlisted for HCC during the “Share 15” period. In 2013, the liver distribution policy was upgraded to a Regional “Share 35” which prioritized regional distribution of livers over local patients [17,23,24]. As an example, from Organ Procurement and Transplant Network (OPTN) Region 5, a liver in San Diego, California would be offered to a MELD 36 patient in San Francisco, California prior to a MELD 34 patient in San Diego itself.

Share 35, while effectively prioritizing high MELD patients, shifted the patterns of organs available for patients with HCC. Until HCC patients accrued MELD > 35, they were subject to higher waitlist mortality and a greater use of extended criteria grafts [25]. Concurrently, there was accumulating evidence of HCC disease stability while on the waitlist due to the use of locoregional therapy, while retaining similar waitlist and post-transplant outcomes across regions with highly variable times to transplant [15,26,27,28]. As a response, in 2015, a revised HCC exception policy was enacted, termed the “Delay and Cap” [29]. This policy attempted to grant HCC patients higher MELD exception points (28) while enforcing a cap of 34 to prevent over-prioritization, while also delaying the provision of exception points for 6-months to help assess tumor aggressiveness, and potentially delist candidates with unfavorable tumor biology [29,30]. 

As HCC grew to account for a growing percentage of the total liver transplants performed with a relatively stable pool of deceased donors in the US, there was deepening concern that not enough livers were being directed to patients with decompensated liver disease. An HCC sponge was developing due to exception points, which was further exacerbated by the criticism that HCC patients were carrying MELD points that far exceeded their risk of waitlist mortality or dropout. In 2019, during this period, HCC policy was shifted to deemphasize transplant access for HCC patients—all HCC patients were given exception points equal to the median MELD at transplant-3 (MMAT-3), calculated from the transplant center of record [31]. This allocated a set number of exception points based on the individual transplant centers’ median MELDs at time of transplant, following a wait period of 6 months. This created an unintended consequence where programs in close geographic proximity may have different exception scores allotted to patients with similar needs for transplantations, and therefore differential access to the highest quality deceased donor livers. To correct this, exception points allotted were changed from the transplant center’s median MELD to the median MELD around the donor hospital on 28 June 2022 [32]. 

**Figure 2:**
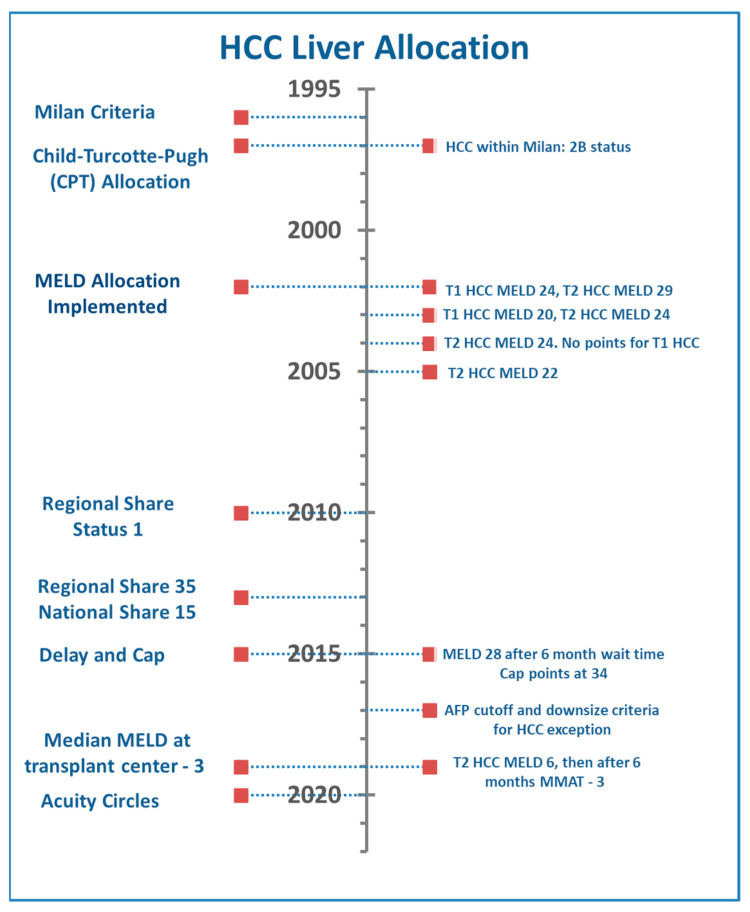
US Liver Allocation Policy and Changes.

All the while, the median MELD at transplant between regions of the US was widening, which created differential waitlist access for patients with the same diagnosis and tumor burden. As an attempt to decrease geographic disparities between transplant centers, the liver distribution system was changed to a concentric circle model around the donor hospital based on acuity—the acuity circles model. In February 2020, the OPTN introduced the acuity circles allocation policy which replaced the prior donation service areas (DSA) and regional boundaries. Allocation was now based on the distances between the donor hospital and transplant center in nautical miles [33]. Early review of the allocation of organs for HCC patients with exception points has suggested lower donation after brain death offer rates while similar or higher donation after circulatory death offer rates [34]. The progression and changes to the US HCC allocation system are summarized in Figure 2.

## 3. Limitations of the Milan Model

While national allocation and distribution policy changes have shifted to deemphasize HCC access to liver transplant, many clinicians have sought to expand access to transplant for patients with HCC beyond Milan criteria. Despite an average acceptable patient survival following implementation of the Milan criteria, there was concern the criteria might be too limiting, with patients who would potentially benefit from a transplant being inappropriately excluded. This prompted studies over the selective expansion of inclusion criteria for liver transplant for HCC. 

Several studies have looked at expanding the Milan Criteria. In 2001, Yao et al. conducted a retrospective evaluation of 70 patients who underwent liver transplantation for HCC. Their explanted livers were examined for tumor burden and outcomes were evaluated based on tumor extent. Based on this study, they established the UCSF Criteria [single nodule ≤ 6.5 cm, or 2–3 tumors (none exceeding 4.5 cm with a tumor sum diameter ≤ 8 cm)] and reported a patient survival equivalent to that of the Milan Criteria with 75% survival at five years [35]. Further evaluation by the UCSF group found that the patients excluded by Milan but within UCSF criteria had a 2-year survival of 86% (95% CI, 54% to 96%) [36]. The UCSF Criteria showed that the Milan Criteria may be modestly expanded without negatively affecting patient outcomes or wasting liver allografts on futile endeavors. These criteria aimed to broaden the pool of potential transplant candidates [36].

Other studies outside the United States paralleled this intent to expand the Milan Criteria. Mazzafero’s group further built on their previous work by developing the ‘up to 7’ criteria in 2009. These were developed from a study of 283 patients without microvascular invasion, but who fell within the Up-to-seven criteria (hepatocellular carcinomas with seven as the sum of the size of the largest tumor [in cm] and the number of tumors) and achieved a 5-year overall survival of 71.2% [37]. 

In response to imaging only criteria, in 2011, Dubay et al. proposed the Toronto Criteria, which incorporated imaging findings to rule out vascular invasion, as well as pathologic criteria to rule out poorly differentiated tumors from transplant [38]. The Toronto Criteria study showed survival was not significantly associated with total tumor size or HCC stage and achieved a 5-year survival of 72% across the entire study which included patients who were beyond Milan criteria. Dubay et al. also exposed a large discrepancy of tumor burden at time of liver explant compared to initial staging imaging. Imaging under-staged 30% of the patients within the Milan group and over staged 23% of the extended criteria group [38]. The inadequacies of imaging staging, the lack of association of tumor size to survival, and the concern that imaging did not predict tumor biology has called for better, more comprehensive, tools to be used for HCC prioritization within the liver allocation scheme.

## 4. HCC, AFP & Liver Transplant

### 4.1. AFP in the Pre-Operative Setting, Allocation and Down-Staging

#### 4.1.1. Survival and Recurrence 

Pre-transplant AFP levels have been shown to be independent predictors of survival and disease recurrence in patients undergoing liver transplantation for HCC. In a 2001 retrospective analysis of 70 patients, Yao et al. found AFP levels > 1000 ng/mL served as an independent predictor of mortality with a hazard ratio of 2.96, independent of whether patients were within Milan Criteria [35]. For the majority of the US, except under certain protocols, the absolute preoperative value of AFP > 1000 ng/mL has been utilized as a red line due to the high risk of recurrence and mortality [14,39,40,41]. Hameed et al. 2014 established that by implementing a cutoff of patients with preoperative AFP > 1000 ng/mL they would exclude only 4.7% of patients from being eligible for transplant, while gaining a 20% reduction in post transplantation HCC recurrence [14]. This preoperative cutoff of AFP > 1000 ng/mL was adopted by the US allocation criteria in 2017, except under region-based protocols, such as the Region 5 down-staging for “all comers” with HCC [42,43].

Although a pre-operative level of 1000 ng/mL appears to be a prohibitive cutoff, there have been multiple studies identifying adverse outcomes associated with lower AFP levels [13,38,44,45,46,47]. In 2009, a large review of more than 6000 patients in the SRTR database confirmed AFP was an independent predictor of survival with a recommended cutoff of 400 ng/mL for access to liver transplant [44]. The Toronto group published a study demonstrating a preoperative AFP > 500 ng/mL as a predictor of poor outcomes with 10 year patient follow-up [45]. A US study, one of the largest United Network for Organ Sharing (UNOS) reviews of over 6000 HCC patients within Milan Criteria, found that 5-year survival progressively decreased as AFP increased, with a measurable survival discrimination with an AFP nadir of 15 ng/mL for 5-year survival outcomes (5-year survival: AFP < 15 ng/mL 74%, AFP 16–65 ng/mL 66.1%, AFP > 65 ng/mL 57.4%) [48]. 

Lower AFP has also been correlated with lower rates of post-transplant recurrence and survival, irrespective of Milan criteria. In select patients exceeding Milan criteria, those with AFP < 100 ng/mL could obtain a 5-year risk of recurrence of only 14.4% vs. 47.6%, *p* = 0.006 [40]. While exact AFP cutoff values demonstrating the best post-transplant outcomes are not exact, AFP < 15 ng/mL at transplant had similar outcomes irrespective of whether the tumor burden was within or beyond Milan Criteria [48]. 

#### 4.1.2. AFP Dynamics 

In addition to the absolute static preoperative value of AFP, there has been evaluation of the dynamic changes of AFP prior to transplantation in response to preoperative therapies and overall post-transplant outcomes. One of the first studies reviewed 153 patients undergoing liver transplantation for HCC (78% underwent locoregional therapy). The first and last AFP points over time were used to generate an AFP slope of progression and found that AFP slope > 15 ng/mL/month had poorer survival (54% vs. 76% *p* = 0.02) at 5 years [49]. Interestingly, in this study, neither static preoperative AFP levels nor Milan criteria reached statistical significance for predicting postoperative recurrence or survival. In a larger review of 336 patients undergoing liver transplantation (98% of whom had preoperative locoregional treatment), Giard et al. established an AFP slope > 7.5 ng/mL/month had a 3-fold higher relative risk of recurrence, which was also strongly associated with microvascular invasion (OR 6.8, *p* = 0.008) [50]. 

A complicating issue in studies of AFP dynamics and determination of AFP slope over time is reliability of AFP measures in the setting of variable locoregional therapies, a lack of accounting for viral hepatitis status, and other issues [4]. It is unclear what threshold of AFP slope is definitively associated with poor outcomes, and wide ranges of positive AFP slopes have been associated with poor outcomes [50,51,52]. It is clear from a clinical standpoint, that tumors that continue to express high levels of AFP despite locoregional therapy have concerning tumor biology, which may warrant more aggressive locoregional therapy, consideration of systemic therapies, as well as avoidance of liver transplantation. There are no uniform practice guidelines related to how to utilize AFP dynamics. Clinicians do not have reliable indicators of ideal AFP slope that correlated with post-transplant outcomes. AFP slope can range widely, with regard to method of calculation as well as final values, which leads to uncertain clinical correlations [50,51,52]. Like the static preoperative AFP, the exact AFP slope values that are relevant is subject of debate.

#### 4.1.3. Down-staging and Allocation

Down-staging is the application of pre-transplant therapies, typically locoregional liver-directed therapy, to decrease the size and number of liver lesions to meet acceptable criteria for transplantation [53]. In 2017, the UCSF downstaging criteria were adopted by UNOS as the upper tumor burden limit for patients eligible for down-staging, with the exception of patients falling under regional protocol variances. Patients meeting the down-staging UCSF criteria (single tumor > 5 and ≤ 8 cm in diameter, 2–3 tumors each ≤ 5 cm in diameter with a sum of all tumors ≤ 8 cm, or 4–5 lesions each < 3 cm sum of all tumors ≤ 8 cm and no evidence of vascular invasion) have been established to achieve similar post-transplant outcomes once down staged to within Milan as compared to patients always within Milan criteria [54]. 

These criteria were supported by a recent UNOS database review (n = 3819), comparing groups always within Milan, with those down-staged per UNOS / UCSF down-staging criteria (UNOS-DS), and those with initial tumor burden beyond UNOS criteria [55]. Although the post-transplant 3-year survival was comparable between the Milan and UNOS-DS groups (83.2% vs. 79.1% *p* = 0.17), within the downstaging groups, AFP ≥ 100 ng/mL at the time of transplant (HR 2.4, *p* = 0.009) and short wait-list region (HR 3.1, *p* = 0.005) were associated with increased risk of post-transplant death. Only AFP ≥ 100 ng/mL proved to be an independent predictor of HCC recurrence [55]. This study supported the current placement of upper limits on tumor burden amenable to downsizing but perhaps more importantly also suggested further evaluation of AFP’s role in prognosticating post-transplant outcomes in down staged patients.

An SRTR database investigation of 6817 patients with a diagnosis of HCC followed the trend of AFP after downstaging treatment. They found that patients with AFP levels originally > 400 ng/mL (even as high as > 1000) who had sufficient treatment response to reduce AFP ≤ 400 ng/mL had similar intention-to-treat and post-transplant survival to patients with AFP always ≤ 400 ng/mL (81% vs. 74% at 3 years, *p* = 0.14 and 89% vs. 78% at 3 years, *p* = 0.11, respectively) [13]. 

In a similar vein, Grat et al. found that patients with AFP persistently < 100ng/mL (97.3%) and those whose AFP dropped below 100 ng/mL (100%) after locoregional treatment, had significantly better 5-year recurrence-free survival compared to those whose AFP rose from <100 ng/mL (75%) or was always >100 ng/mL (38.4%) (*p* < 0.001) [56]. These studies suggested a link between AFP response to downstaging and post-transplant recurrence-free survival. 

In 2017, the US Allocation system formally adopted an AFP cutoff of 1000 ng/mL to qualify for HCC exception points. If AFP > 1000 ng/mL, the patient would be required to downstage to an AFP < 500 ng/mL and stay <500 ng/mL for 3 months prior to qualification for exception points [53]. The AFP response to therapy, as a result of this policy, would provide a more precise measurement of tumor biology over time as compared to the initial fears of recurrence from the static initial AFP value of 1000 ng/mL, as previously discussed [14]. 

#### 4.1.4. Waitlist Mortality and Dropout

Waitlist mortality and dropout have been a long subject of concern given the historical difficulty of adopting an equitable allocation system. More recent alterations to the US allocation system included a 6-month waitlist period and exception point cap in 2015 to address discrepancies on a national level, and to encourage selection of transplant candidates with favorable tumor biology [57]. Median MELD at Transplant of the transplant center minus three points (MMAT-3) was selected as the exception point score for HCC patients in 2019. Although enacted only recently, a large UNOS database review of dropout since the MMAT-3 policy has found dropout for both non-HCC (from 12.9% to 11.1%) and HCC (from 14% to 10.7%) patients have begun to normalize, suggesting a more equitable allocation model compared to prior [57]. In 2022, this policy was recently modified so that MMAT would be calculated around the donor hospital rather than the transplant hospital in order to provide relative equal access to transplant for patients in geographically contiguous areas at centers with vastly different median MELDs at transplant. The results of this model of allocation for HCC yields a dynamic MELD score for exception patients based on different match runs from different donors. The same patient may receive a transplant with different exception MELD scores at transplant depending on the origin of the donor organ. The results of this policy are maturing, but they hold significant promise in equitably allocating livers with HCC across the country.

While this is encouraging, there is continued concern that certain HCC patients are being inappropriately prioritized. All HCC patients are currently given the same allocation priority irrespective of their liver dysfunction or tumor biology; therefore, patients at a low risk for dropout are given the same priority as those with high risk. Precision in identifying higher dropout risk patients remains lacking in the current allocation scheme. 

Current studies have identified risk factors for dropout, but these studies lack uniformity in access to donor organs. Known risk factors for patient dropout while on the waitlist include a high AFP at time of listing, rapid rise in AFP, lack of response to locoregional therapy and synthetic liver dysfunction [13,54,58,59]. The exact value of static listing AFP level associated with dropout varies widely per study, with ranges from 20 to 400 ng/mL quoted [13,60]. Pre-treatment AFP of >500 ng/mL has also been found to predict dropout, independent of Milan status [45].

AFP has evolved in the downstaging space. In addition to serial evaluation by MRI to look for imaging characteristics consistent with tumor viability, AFP trajectory has been used to evaluate response to therapy. For patients undergoing down-staging with locoregional therapy, in the setting of originally high AFP, dropout risk may be reduced with therapy to the level of those patients whose original AFP was lower and persisted. Merani et al. found a similar dropout rate of 10% in patients with AFP either always <400 ng/mL or who fell below <400 ng/mL following locoregional therapy. Those who were either always over 400 or who rose to > 400 ng/mL had a significantly increased risk of dropout at 25% and 44%, respectively, (*p* < 0.001) [13]. 

A more recent, large-scale analysis found factors predicting dropout following downstaging pretreatment AFP ≥ 1000 ng/mL (multivariate hazard ratio [HR]: 2.42; *p* = 0.02) and Child’s B versus Child’s A cirrhosis (multivariate HR: 2.19; *p* = 0.04) [54]. Mehta et al. identified the following factors predicting low dropout rates: AFP < 20 ng/mL, MELD < 15, child’s class A, and single 2–3 cm lesions [60]. This was further validated into a risk score in 2021, with static AFP being the most heavily weighted [57].

### 4.2. Utilization of AFP following Liver Transplantation

Despite optimization and Milan criteria, HCC recurrence post-transplant still occurs in 10–20% of cases [61]. Most recurrences present with extrahepatic disease (78.1%) and are associated with a median survival of 10 months despite treatment (95% CI, 6.5–15.7 months) [61].

Patients with recurrence have better survival when the disease is amenable to resection, locoregional therapy, and more recently immunotherapy [61,62,63]. As such, early detection of recurrence is imperative to optimize available therapy. Although some centers have post-transplant surveillance protocols, there is no universal post-transplant protocol for screening patients, how to use AFP as a biomarker, or to determine which patients need more intensive surveillance [64].

As previously discussed, preoperative AFP and AFP slope have been shown to be independent predictors of post-transplant HCC recurrence. A large UNOS database review of patients with post-transplant recurrence found a preoperative AFP > 500 ng/mL was also linked to lower recurrence-free survival. Preoperative elevated AFP also proved to be an independent risk factor for survival among recurrent HCC patients, with a 1.6-fold increased risk of death when compared to those with preoperative AFP < 20 ng/mL [65].

In addition to the preoperative AFP, the post-transplant AFP trend can serve as an indicator of recurrence risk. In a retrospective review of 125 patients with elevated preoperative AFP (>20 ng/mL) undergoing liver transplant, patients who had rapid AFP normalization within one-month post-transplant had less tumor recurrence. Non-rapid normalization served as a risk factor, independent of Milan criteria status, for recurrence with a hazard ratio of 4.41, *p* < 0.001 [66]. Utilization of the pre- and post-AFP trends could be useful in developing protocols for recurrence risk and postoperative monitoring. 

## 5. Prediction Models for Post-Transplant Survival in HCC: The Role of AFP 

**Figure 3:**
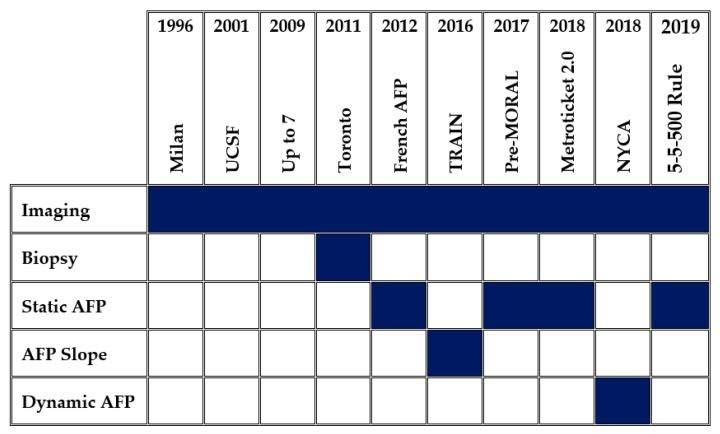
HCC Prediction Models over time. As time has progressed, a greater focus on AFP inclusion is noted in developing prediction models for recurrence-free survival in the setting of liver transplantation.

Initial prediction models of post-transplant survival for HCC, including those for selection, utilized imaging-based criteria. As time progressed, several groups began to incorporate different forms of AFP to augment the predictive validity of their models (Figure 3). Metroticket 2.0 and Pre-MORAL incorporated preoperative AFP static numbers to predict post-operative survival and recurrence free survival [67,68]. RETREAT is a post-operative recurrence risk score developed by the UCSF group that incorporates the explant pathology in addition to the last preoperative AFP [64]. The 5-5-500 Rule utilizes imaging and static AFP; since 2019 it has served Japan as the insurance covered, national selection criteria for HCC liver transplant candidates [69].

A newly proposed, and recently validated NYCA criteria incorporates the AFP response during downstaging and time on waitlist to help establish a recurrence-free survival risk [70]. In their score, dynamic AFP was the highest weighted factor in future recurrence. After the initial proposal of the score in 2018, it was externally validated and found to significantly better predict recurrence when compared to current leading HCC prediction models [71]. A list of the more common prediction models is summarized in Table 1.

## 6. Conclusions

AFP is the most frequently utilized biomarker in the clinical management of HCC. Present in 60–80% of patients, circulating serum AFP levels have demonstrated clinical utility, as it corresponds with tumor expression and growth. It has demonstrated utility for candidate selection, is a proxy of tumor response to therapy, aids in prognostication of tumor recurrence after transplantation. For clinicians, the incorporation of AFP is deeply ingrained in the daily clinical practice of identifying candidates for locoregional therapy, selection of candidates for the liver wait-list, and identification of patients with aggressive tumors where there is little transplant benefit. 

Since the adoption of the Milan Criteria, the US allocation of HCC patients has undergone extensive revisions to improve equity and to minimize post-transplant recurrence. Despite over two decades of alterations, however, the policy remains largely dependent on Milan criteria, limited in AFP utilization, and unable to stratify HCC patients based on waitlist dropout risk. The Milan criteria created a paradigm based on imaging criteria which are now known to be an imperfect proxy of tumor biology. Imaging is often inconsistent with final explant pathology, suggesting it is an unreliable marker of tumor biology. Multiple studies have achieved similar post-transplant recurrence rates with patients outside Milan when other variables have been met (low AFP, no vascular invasion, compensated liver disease, lack of undifferentiated histology) [35,38,70]. Yet, the Milan criteria remains pivotal to acquiring HCC exception points and accruing a competitive MELD score. 

This is further troubling due to the inability to discriminate HCC patients by their tumor biology and dropout risk in the current paradigm. Patients with aggressive tumors are treated similar to patients with more indolent growth patterns. Should a patient whose AFP rises from 100 to 800 ng/mL during their 6 month wait period be granted the same exception points? Is a patient whose AFP continues to uptrend despite undergoing maximum therapy to be considered the same as one who had AFP response? Despite the growing evidence on the utility of AFP as a post-transplant HCC recurrence prognosticator, its widespread incorporation in the allocation policies has been slow to occur. It was not until 2017 that static AFP was incorporated into the UNOS allocation policy. More recent work published by, Halazun et al. to create the NYCA score has demonstrated that dynamic AFP response (NYCA score) to locoregional treatment is a better prognostic tool of HCC recurrence compared to static AFP, which only provides a pre-transplant AFP snapshot. Utilization of the NYCA score for candidate selection tremendously expands the proportion of patients hitherto deemed non-transplantable due to high static AFP/ tumor burden, without compromise to recurrence-free survival [70,73]. The NYCA scoring system incorporates the preoperative AFP response to stratify patients into low, acceptable, and high risk for 5-year recurrence, irrespective of their Milan status. Despite broad external validation [70], the current UNOS allocation policies are yet to incorporate dynamic AFP into the UNOS allocation model. 

This is particularly important in the US, where most HCC patients receive deceased donor liver allografts. The current U.S. liver distribution system has prioritized broader organ sharing and lower priority for HCC patients. For HCC patients both currently meeting exception score criteria as well as those beyond criteria (but with favorable characteristics), centers are increasingly limited by deceased donor availability, which risks progression of disease and waitlist dropout, or the utilization of extended criteria grafts which may impact outcomes independent of tumor biology. Clinicians need uniform national policies guided by the biology of the tumor of the patient in front of them. This is particularly important as HCC-directed therapies evolve, with improving technology for locoregional therapy and monumental shifts in available systemic therapies [74]. 

In summary, the US transplant community should revisit its HCC organ allocation model and incorporate more precise oncologic principles to select patients for transplant. Strong consideration should be given to incorporating AFP dynamics to enable waitlist dropout risk stratification. This will improve candidate selection and likely expand the pool of patients who could benefit from transplant, without compromising the optimal derived societal beneficence from available organs. 

## Figures and Tables

**Table 1:** Liver transplant for HCC prediction models. Summarizes the criteria and findings for each prediction model. Neutrophil-lymphocyte ratio (NLR), platelet-lymphocyte ratio (PLR), Modified response evaluation criteria in solid tumors (mRECIST).

Study	Basis	No of Patients	Type	Findings
Milan Criteria (Mazzaferro 1996) [6]	Imaging	48	Largest tumor < 5 cm, or No more than 3 tumor nodules, each <3 cm, and No obvious vessel or nodal involvement	4-year survival: 75% 4-year recurrence-free survival: 83%
UCSF (Yao 2001) [35]	Imaging	70	Single tumor ≤ 6.5 cm, or ≤3 tumors Largest ≤ 4.5 cm diameter and Total tumor diameter ≤ 8 cm	5-year survival: 75.2%
Up-to-7 (Mazzaferro 2009) [37]	Imaging	283	Sum of the diameter (cm) of largest tumor and the number of tumors ≤ 7 Excluded microvascular invasion	5-year survival: 71.2%
Extended Toronto (DuBay 2011) [38]	Imaging and Biopsy	105	No tumor size or number limit No systemic spread or vascular involvement Not poorly differentiated on biopsy (if exceeds Milan)	5-year survival: 70% 5-year disease-free survival: 66%
AFP Model (Duvoux 2012) [40]	Imaging and static AFP	537	Tumor largest diameter (≤3, 3–6, >6 cm) Number of tumors (1–3, 4) At listing AFP (≤100, 101–1000, >1000 ng/mL)	5-year overall survival: Low risk: 69.9% High risk: 40.8%
TRAIN Score (Lai 2016) [72]	Imaging and AFP slope	179	mRECIST (response or no) AFP slope (>15 ng/mL/month) NLR and PLR Waitlist time	5-year survival: TRAIN < 1.0: 67.5% 5-year recurrence: TRAIN < 1.0: 8.9%
Pre-MORAL (Halazun 2017) [68]	Imaging, NLR, static AFP		NLR > 5 AFP > 200 ng/mL Tumor size > 3 cm	Recurrence-free survival (RFS) 5 year: Low risk: 98.6% Medium risk: 69.8% High risk: 55.8%
Metroticket 2.0 (Mazzaferro 2018) [67]	Imaging and static AFP	1018	Up-to-7 criteria Last pre-op AFP (<200; 200–400; 401–1000; >1000 ng/mL)	5-year overall survival: 79.7% If within Green area
NYCA (Halazun 2018) [70]	Imaging, AFP response, NLR	1450	At time of diagnosis: Maximum tumor size Maximum tumor number AFP response AFP always < 200 ng/mL Responders Non responders	5-year recurrence-free survival Score: Low 0–2: 90% Acceptable 3–6: 70% High ≥ 7: 42% AFP non-responders had the greatest points allocated.
RETREAT (Mehta 2017) [64]	Explant pathology AFP static		On Explant: Tumor burden Largest viable tumor diameter Number viable tumors Microvascular invasion AFP at time of transplant	5-year recurrence risk Score: 0: 3% 1: 8% 2: 11% 3: 14% 4: 29% ≥5: 75%
5-5-500 Rule (Shimamura 2019) [69]	Imaging and static AFP	965	For living liver transplant Largest tumor ≤5 cm ≤5 tumors AFP ≤ 500 ng/mL	5-year recurrence risk: 7.3%
